# Natural Orifice Specimen Extraction for Right-Sided Colon Cancer: A Systematic Review and Meta-Analysis of Propensity Score-Matched Studies

**DOI:** 10.7759/cureus.84191

**Published:** 2025-05-15

**Authors:** Bernardo F Pompeu, Victória Mara Vieira Rocha, Ana Flávia Machado Oliveira, Patricia Marcolin, Luís C dos Lucio Generoso, Sérgio Mazzola Poli De Figueiredo, Fernanda B Formiga

**Affiliations:** 1 General and Colorectal Surgery, University of São Caetano do Sul, São Paulo, BRA; 2 Colorectal Surgery, Hospital Heliópolis, São Paulo, BRA; 3 Medicine, Federal University of the Southern Border (UFFS), Chapecó, BRA; 4 Surgery, University of North Carolina at Chapel Hill, Chapel Hill, USA; 5 Colorectal Surgery, Hospital Heliopolis, São Paulo, BRA

**Keywords:** laparoscopic surgery, natural orifice specimen extraction, postoperative outcomes, propensity score matching, right-sided colon cancer

## Abstract

Minimally invasive surgery is the standard approach for right-sided colon cancer, but conventional laparoscopic specimen extraction (CVT) requires additional abdominal incisions, increasing the risk of postoperative complications and delayed recovery. Natural orifice specimen extraction (NOSE) minimizes abdominal incisions, potentially improving patient outcomes. This meta-analysis compares NOSE and CVT in terms of postoperative complications, operative characteristics, and long-term outcomes. A comprehensive literature search was conducted in PubMed, Scopus, the Cochrane Central Register of Clinical Trials, and Web of Science for studies available up to December 2024. A random-effects model was applied to compute ORs and mean differences (MDs) with 95% CIs. Heterogeneity was evaluated using the I² statistic. All statistical analyses were performed using R software (version 4.4.1, R Foundation for Statistical Computing). Seven propensity score-matched studies with 566 patients were included, with 240 (42.4%) undergoing NOSE and 326 (57.6%) undergoing CVT. NOSE was associated with significantly reduced postoperative pain on the 3rd day (MD -1.1; 95% CI -1.7 to -0.5; p < 0.01), lower SSI rates (OR 0.23; 95% CI 0.08-0.73; p = 0.012), and a shorter time to pass flatus (MD -0.8; 95% CI -1.2 to -0.4; p < 0.01). However, NOSE was linked to longer operative times (MD 36.4 minutes; 95% CI 3.4-69.4; p = 0.03). No significant differences were found in hospital stay (MD -0.5 days; 95% CI -2.1 to 1.1; p = 0.57), blood loss (MD -2.1; 95% CI -9.6 to 5.4; p = 0.58), or local recurrence (OR 0.44; 95% CI 0.07-3.01; p = 0.405). In conclusion, NOSE offers advantages such as reduced postoperative pain, lower SSI rates, and faster bowel recovery, with prolonged operative time as its main limitation. These findings support NOSE as a viable alternative to CVT for right-sided colon cancer without compromising safety or long-term outcomes.

## Introduction and background

Colorectal cancer ranks as the third most frequently diagnosed malignancy worldwide and stands as the second leading cause of cancer-related deaths [1,2]. Advances in minimally invasive surgical techniques, particularly laparoscopic-assisted procedures, have revolutionized the management of this malignancy, offering excellent oncological outcomes [3-13]. In right colectomies, anastomosis may be performed either intracorporeally or extracorporeally, depending on the surgeon’s proficiency and experience [14]. Despite the benefits of laparoscopic surgery, conventional approaches still require an abdominal incision of approximately 10 cm for specimen extraction, which may contribute to postoperative pain and delayed recovery [3,6-8,10].

The concept of laparoscopic natural orifice specimen extraction (NOSE) was first introduced in 1993 and has progressively developed into a viable alternative to conventional laparoscopic right colectomy (CVT) [6,15]. This technique enables colonic mobilization, transection, and intracorporeal anastomosis to be performed exclusively through laparoscopic methods, with specimen retrieval occurring via transcolonic, transrectal, or transvaginal routes [3-13]. By eliminating the need for an abdominal incision, NOSE reduces surgical trauma and promotes faster postoperative recovery, marking a significant advancement in minimally invasive surgery [3,6-8,10].

Recent meta-analyses comparing NOSE with conventional laparoscopic approaches for right-sided colon cancer have identified critical gaps in the literature [16]. However, many of the included observational studies were subject to a high risk of confounding bias. Notably, no randomized controlled trials (RCTs) are currently available on this topic, limiting the level of evidence in previous analyses. In light of recently published high-quality studies, an updated analysis is warranted to reassess previous findings [8,9,13]. To enhance methodological rigor and minimize confounding, this meta-analysis exclusively includes studies utilizing propensity score matching (PSM). Notably, the results presented here diverge significantly from those reported in prior meta-analyses, offering new insights into intraoperative, postoperative, and long-term outcomes.

## Review

Materials and methods

This systematic review was conducted in accordance with the Preferred Reporting Items for Systematic Reviews and Meta-Analyses (PRISMA) guidelines [17]. The study protocol was registered in the International Prospective Register of Systematic Reviews (PROSPERO) under registration number CRD42024628840 [18]. Since this research is based on a systematic review and meta-analysis of previously published studies, it does not require ethical approval.

Search Strategy

A comprehensive literature search was conducted in PubMed, the Cochrane Central Register of Clinical Trials, Web of Science, and Scopus, including studies published up to December 2024. The search strategy employed was as follows: (("Colorectal Cancer" OR "Colonic cancer" OR "Right-sided colon cancer" OR "Right colon cancer" OR "Colon neoplasms" OR "Colorectal neoplasms" OR "Colon* carcinoma" OR "Colonic tumor" OR "Right colonic cancer" OR "Right colectomy" OR "Right hemicolectomy" OR "colon* adenocarcinoma" OR "Malignant neoplasm of the colon" OR "Large bowel cancer") AND ("Natural orifice specimen extraction" OR NOSES OR NOSE OR NICE OR "Transrectal specimen extraction" OR "Transvaginal specimen extraction" OR "Transcolonic specimen extraction" OR "Natural orifice surgery" OR "NOTES" OR "Specimen extraction") AND ("Conventional colectomy" OR "Laparoscopic colectomy" OR "Open colectomy" OR "Hemicolectomy" OR "Right hemicolectomy" OR "Transabdominal specimen extraction" OR "Laparoscopic surgery" OR "Surgical specimen extraction" OR "Minimally invasive colectomy" OR "Minimally invasive surgery" OR "Traditional colectomy" OR "Conventional surgery")).

Eligibility Criteria

This study included observational research utilizing propensity score matching (PSM) to compare NOSE with conventional laparoscopic procedures in patients undergoing right-sided colectomies for colon cancer. Exclusion criteria comprised: (1) patients who underwent diverting procedures; (2) studies without a control group; and (3) case reports, conference abstracts, review articles, or animal studies.

Data Extraction and Endpoints

​Two authors (V.M.V.R and A.F.M.O) independently screened the articles for inclusion criteria and extracted data from the selected studies. Any disagreements were resolved by consensus or, if necessary, by consulting a third author (B.F.P). The outcomes assessed were postoperative complications, including: (1) surgical site infection (SSI), (2) visual analog scale (VAS) score at day 3, (3) VAS score at day 1, (4) Clavien-Dindo grade ≥ 3 complications, (5) ileus, (6) urinary retention, (7) UTI, (8) intra-abdominal abscess, (9) anastomotic bleeding, (10) anastomotic leak, (11) operative time, (12) time to first flatus, (13) blood loss, (14) hospital stay, (15) incisional hernia, and (16) local recurrence.

Quality Assessment

Two authors (B.F.P and V.M.V.R) independently assessed the quality of included studies using the Cochrane Collaboration tool for assessing the risk of bias in non-randomized studies (ROBINS-I) [19]. In this assessment, each study was categorized as critical, serious, moderate, or low risk in the seven domains: confounding, selection, classification, deviations from intended interventions, missing data, measurement of outcome, and selection of reported results. Disagreements were resolved unanimously with the senior author (F.B.F).

Statistical Analysis

We calculated pooled ORs for binary outcomes and mean differences (MDs) for continuous variables, both with 95% CIs. A random-effects model was applied for all analyses, with statistical significance set at p<0.05. Heterogeneity was evaluated using the Cochran Q test and I² statistic, with p-values below 0.10 and I² >25% considered indicative of significant heterogeneity. For outcomes with substantial heterogeneity, Baujat plots were employed to determine each study’s influence on the overall effect and heterogeneity. Additionally, leave-one-out sensitivity analyses were performed by sequentially excluding individual studies from the pooled estimates to assess the robustness of the findings. Statistical analyses were conducted using R Software (R Foundation for Statistical Computing), version 4.4.1.

Results

Study Selection and Characteristics

**Figure 1:**
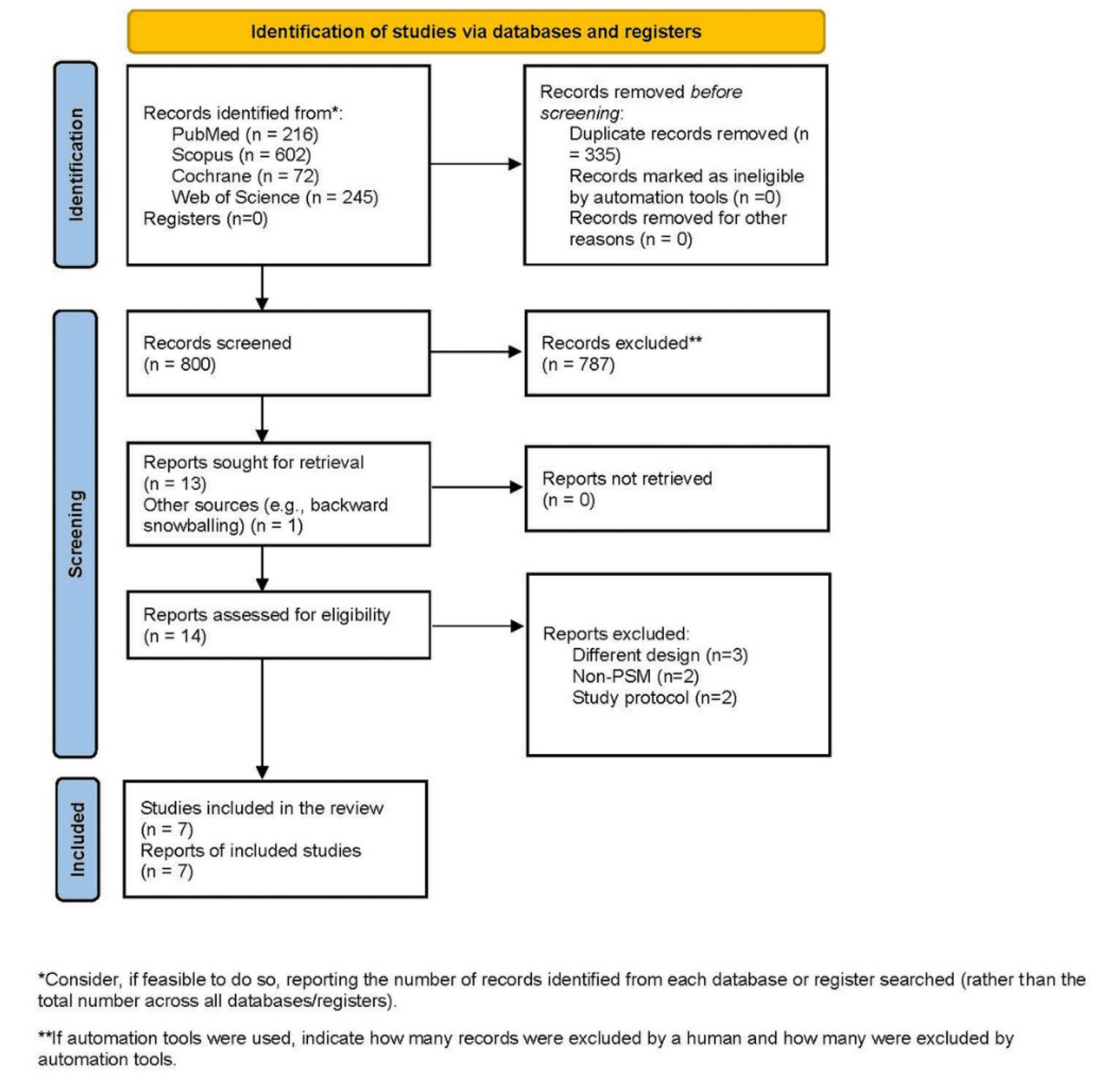
PRISMA flow diagram of study screening and selection. PRISMA: Preferred Reporting Items for Systematic Reviews and Meta-Analyses.

As shown in Figure 1, the initial database search identified 1,135 records. After screening, 335 duplicates were removed and 787 records were excluded based on titles and abstracts, leaving seven observational studies using PSM for the final analysis [3,8-13]

The included trials involved 566 patients diagnosed with colon cancer who underwent right colectomy. Of these, 240 (42.4%) underwent NOSE, while 326 (57.6%) received CVT [3,8-13]. Women constituted 64.66% of the study population. The average age was 61.38 ± 11.61 years in the NOSE group and 61.37 ± 11.61 years in the CVT group [3,8-13]. The mean BMI was 22.95 ± 3.68 kg/m² in the NOSE group and 23.65 ± 3.68 kg/m² in the CVT group [3,8-13].

In the included trials, specimen extraction methods were distributed as follows: 54.58% via the vaginal route, 18.75% via the transcolonic route, and 26.67% via the rectal route [3,8-13]. In contrast, the conventional laparoscopic group utilized laparotomy incisions for specimen extraction [3,8-13]. Tumor localization was reported as 37.58% in the cecum, 38.93% in the ascending colon, 18.46% in the hepatic flexure, and 5.03% in the proximal transverse colon [3,8-13]. TNM staging indicated that 32.31% of cases were classified as stage I, 37.42% as stage II, and 30.27% as stage III. According to T-TNM classification, 49.26% of tumors were in the T1-T2 stages and 50.74% in the T3-T4 stages [3,8-13]. The average follow-up period was 32.52 ± 7.72 months for the NOSE group and 38.35 ± 7.70 months for the CVT group [3,8-13]. Additional details regarding study characteristics are provided in Tables 1-2.

**Table 1:** Baseline characteristics of the observational studies included in the analysis. R-PSM: Retrospective propensity score-matched; P-PSM: Prospective propensity score-matched; NA: Not available or not applicable; NOSE: Natural orifice specimen extraction for laparoscopic right colectomy; CVT: Conventional laparoscopic right colectomy.

Author	Country	NOSE / CVT	Design	Sex (Female), n (%) NOSE / CVT	BMI (kg/m²), Mean ± SD NOSE / CVT	Age (Years), Mean ± SD NOSE / CVT	ASA n (%) NOSE / CVT	Tumor Location n (%) NOSE / CVT
Awad ZT and Griffin R (2014) [3]	USA	20 / 20	R-PSM	20 (100) / 20 (100)	25.1 ± 6.65 / 31.6 ± 8.33	66.9 ± 8.9 / 63.6 ± 9.08	I: 0 (0) / 1 (2.5) II: 4 (10) / 4 (10) III: 15 (37.5) / 15 (37.5) IV: 1 (2.5) / 0 (0)	NA
Kong FB et al. (2021) [12]	China	45 / 45	R-PSM	27 (60.0) / 23 (51.1)	20.0 ± 2.1 / 21.2 ± 2.5	56.9 ± 11.7 / 57.7 ± 14.6	I: 9 (20.0) / 5 (11.1) II: 34 (75.5) / 37 (82.2) III: 2 (4.5) / 3 (6.7)	NA
Li XW et al. (2019) [11]	China	31 / 31	R-PSM	31 (100) / 31 (100)	23.7 ± 3.9 / 25.5 ± 4.7	70.0 ± 9.2 / 68.9 ± 11.9	I: 8 (25.8) / 9 (29.0) II: 17 (54.8) / 19 (61.3) III: 6 (19.4) / 3 (9.7)	Cecum: 8 (25.8) / 7 (22.6) Ascending: 15 (48.4) / 13 (41.9) Hepatic flexure: 8 (25.8) / 11 (35.5)
Park JS et al. (2011) [10]	Korea	34 / 34	R-PSM	34 (100) / 34 (100)	23.9 ± 3.1 / 23.1 ± 2.7	61.0 ± 11.2 / 63.6 ± 11.6	I: 12 (35.3) / 14 (41.1) II: 18 (53.0) / 14 (41.1) III: 4 (11.7) / 6 (17.8)	Cecum: 10 (29.5) / 8 (23.5) Ascending: 16 (47.0) / 19 (55.9) Proximal transverse: 8 (23.5) / 7 (20.6)
ReDati D et al. (2024) [8]	China	24 / 24	R-PSM	3 (12.5) / 5 (20.8)	22.9 ± 3.0 / 23.2 ± 3.1	59.5 ± 2.1 / 59.8 ± 2.3	I-II: 23 (95.8) / 22 (91.7) III: 1 (4.2) / 2 (8.3)	Cecum: 12 (50.0) / 13 (54.2) Ascending: 8 (33.3) / 7 (29.2) Hepatic flexure: 4 (16.7) / 4 (16.7)
Yu H et al. (2023) [9]	China	46 / 92	R-PSM	46 (100) / 92 (100)	24.0 ± 3.4 / 23.2 ± 3.4	58.9 ± 12.5 / 60.3 ± 13.8	I-II: 11 (23.9) / 23 (25.0) III: 35 (76.1) / 69 (75.0)	NA
Zhang M et al. (2023) [13]	China	40 / 80	R-PSM	0 (0) / 0 (0)	22.6 ± 3.6 / 23.2 ± 2.8	61.3 ± 9.7 / 60.7 ± 12.6	NA	Cecum: 20 (50.0) / 34 (42.5) Ascending: 12 (30.0) / 26 (32.5) Hepatic flexure: 8 (20.0) / 20 (25.0)

**Table 2:** Surgical characteristics of the studies included in the meta-analysis. * Median (no range given); NA: Not available or not applicable; NOSE: Natural orifice specimen extraction for laparoscopic right colectomy; CVT: Conventional laparoscopic right colectomy.

Author	T of TNM n (%) NOSE / CVT	TNM Stage n (%) NOSE / CVT	Specimen Extraction (NOSE) n (%)	CVT Extraction	Conversion n (%) NOSE / CVT	Type of Anastomosis	Follow-up (months), Mean ± SD NOSE / CVT
Awad ZT and Griffin R (2014) [3]	NA	I: 8 (20) / 2 (5) II: 2 (5) / 5 (12.5) III: 4 (10) / 4 (10)	Vaginal 20 (100)	4-cm periumbilical incision	NA	Antiperistaltic, Side-to-Side (Stapled)	31.7 ± 9.6 / 51.2 ± 9.6
Kong FB et al. (2021) [12]	T1: 30 (66.7) / 31 (68.9) T2: 10 (22.2) / 8 (17.8) T3: 5 (11.1) / 6 (13.3)	I: 36 (80.0) / 35 (77.8) II: 6 (13.3) / 7 (15.5) III: 3 (6.7) / 3 (6.7)	Transcolonic 45 (100)	5-cm periumbilical incision	NA	Antiperistaltic, Side-to-Side (Stapled)	28.1 ± 4.0 / NA
Li XW et al. (2019) [11]	T1: 8 (25.8) / 8 (25.8) T2: 14 (45.2) / 14 (45.2) T3: 9 (29.0) / 9 (29.0)	NA	Vaginal 31 (100)	NA	0 (0) / 0 (0)	NA	NA
Park JS et al. (2011) [10]	T1: 4 (11.8) / 6 (17.6) T2: 8 (23.5) / 6 (17.6) T3: 22 (64.7) / 22 (64.7)	I: 11 (32.3) / 11 (32.3) II: 16 (47.0) / 14 (41.2) III: 7 (20.6) / 9 (26.5)	Vaginal 34 (100)	5–7 cm transabdominal incision	2 (5.9) / 0 (0)	Antiperistaltic, Side-to-Side (Stapled)	22.8 ± 8.3 / 25.5 ± NA
ReDati D et al. (2024) [8]	T1–T2: 15 (62.5) / 13 (54.2) T3–T4: 9 (37.5) / 11 (45.8)	I: 6 (25.0) / 6 (25.0) II: 13 (54.2) / 12 (50.0) III: 5 (20.8) / 6 (25.0)	Rectal 24 (100)	5–8 cm transabdominal incision	0 (0) / 0 (0)	Isoperistaltic, Side-to-Side (Stapled)	31.5 *
Yu H et al. (2023) [9]	T1: 5 (10.9) / 11 (12.0) T2: 4 (8.7) / 5 (5.4) T3: 22 (47.8) / 34 (37.0) T4: 15 (32.6) / 42 (45.7)	I: 8 (17.4) / 14 (15.2) II: 20 (43.5) / 33 (35.9) III: 18 (39.1) / 45 (48.9)	Vaginal 46 (100)	Median abdominal incision	NA	Isoperistaltic, Side-to-Side (Stapled)	37.7 ± 7.4 / 41.6 ± 8.5
Zhang M et al. (2023) [13]	NA	I: 6 (15.0) / 15 (18.8) II: 18 (45.0) / 37 (46.3) III: 16 (40.0) / 28 (35.0)	Rectal 40 (100)	Vertical periumbilical or Pfannenstiel incision	0 (0) / 1 (0.9)	End-to-Side or Antiperistaltic, Side-to-Side (Stapled)	37.9 ± 5.6 / 35.1 ± 5.0

Pooled analyses of all studies

Postoperative Complications

**Figure 2:**
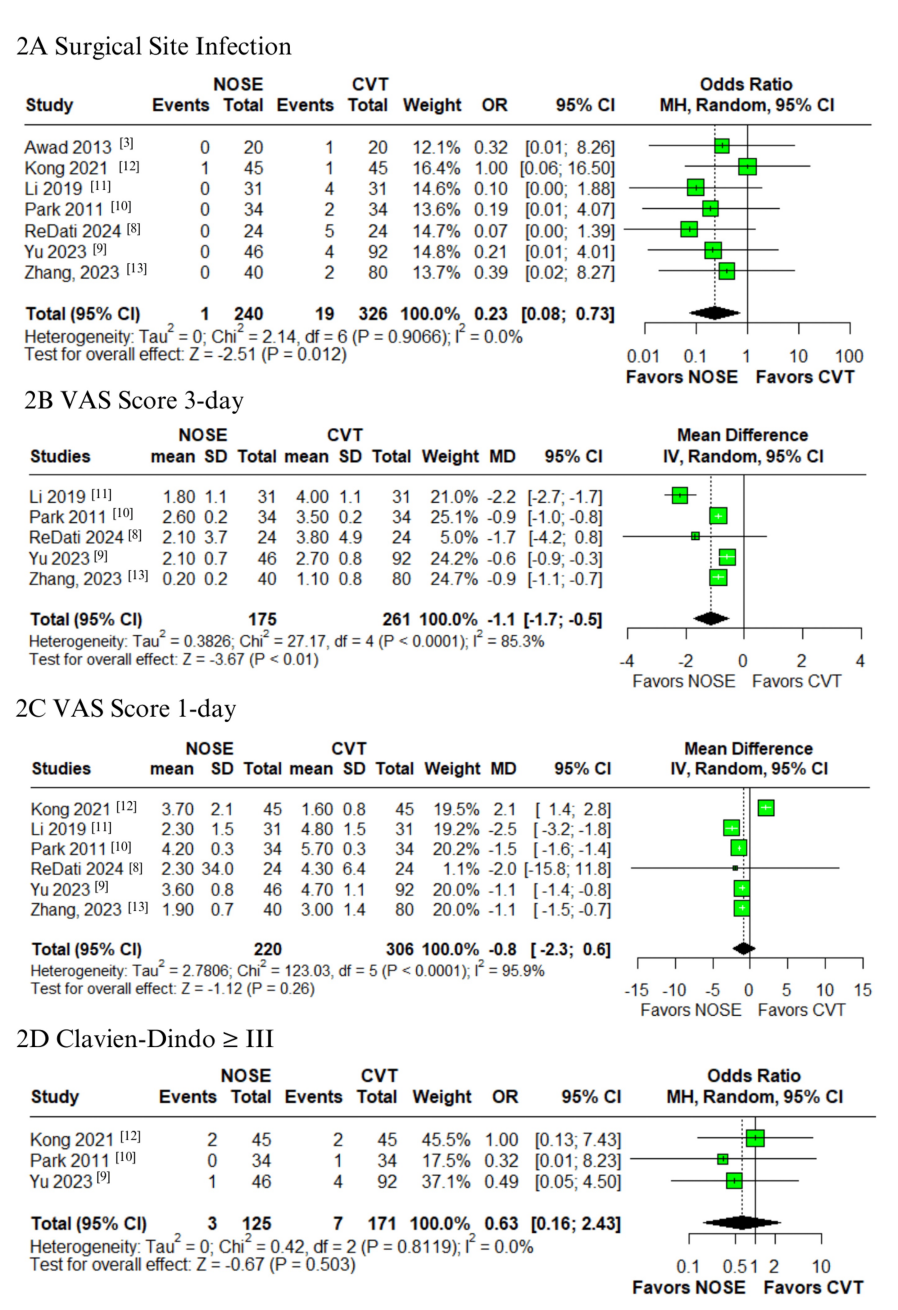
Forest plots comparing NOSE versus conventional laparoscopic surgery in right-sided colon cancer: (A) Surgical site infection (SSI), (B) Visual analog scale (VAS) score on postoperative day 3, (C) VAS score on postoperative day 1, and (D) Clavien-Dindo grade ≥ III complications. NOSE: Natural orifice specimen extraction; SSI: Surgical site infection; VAS: Visual analog scale.

**Figure 3:**
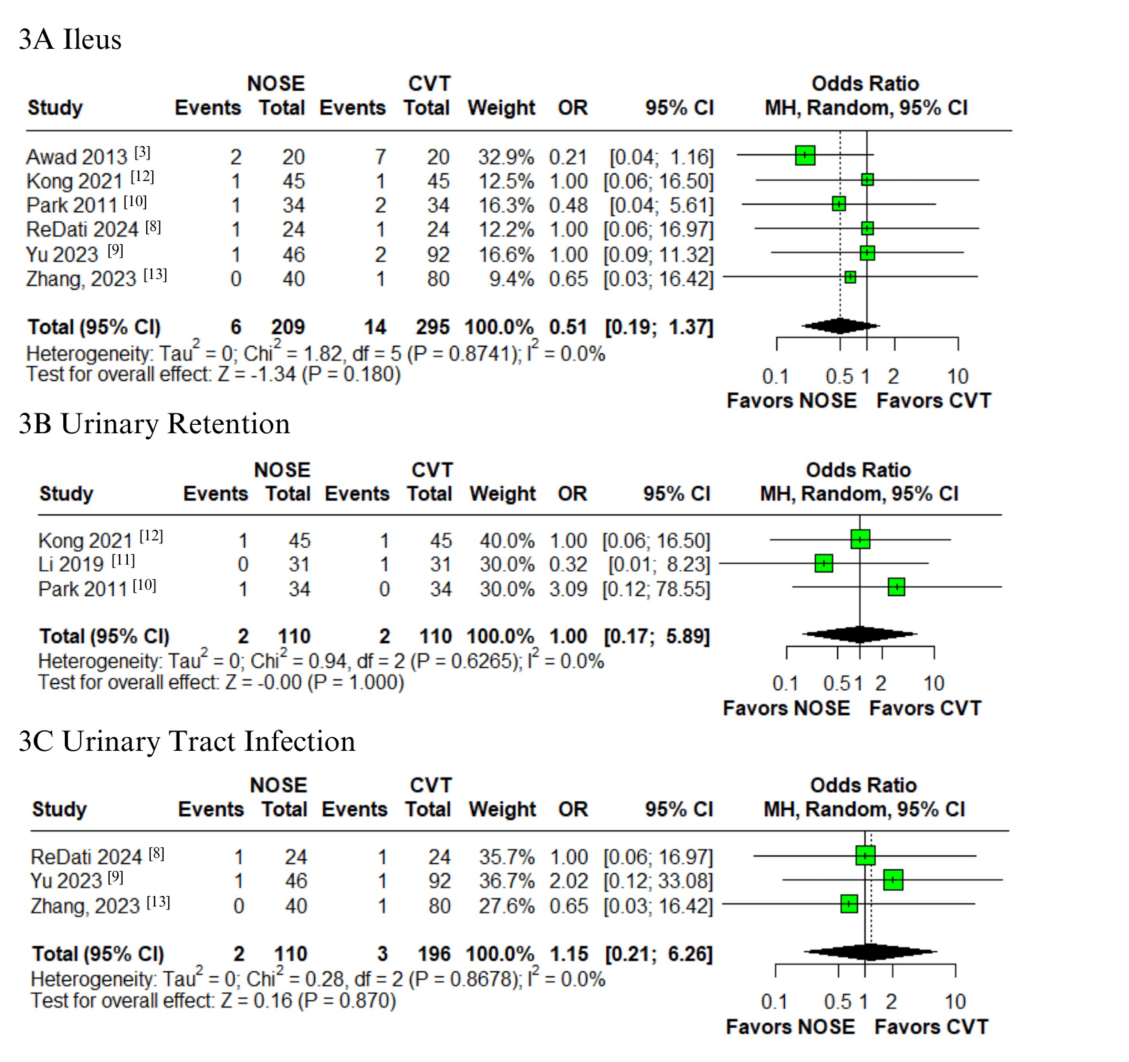
Forest plots comparing NOSE versus conventional laparoscopic surgery in right-sided colon cancer: (A) Ileus, (B) Urinary retention, and (C) UTI. NOSE: Natural orifice specimen extraction.

**Figure 4:**
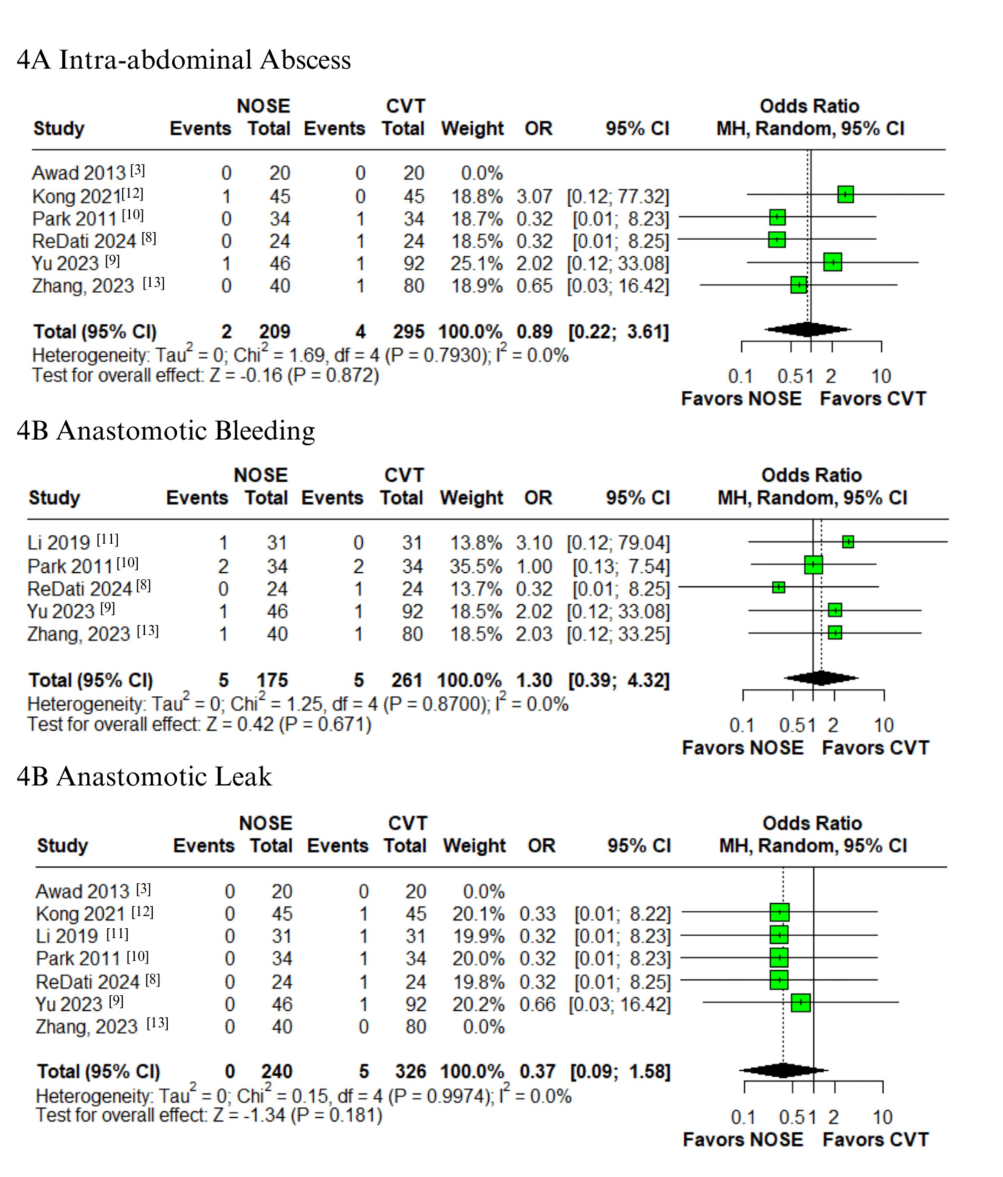
Forest plots comparing NOSE versus conventional laparoscopic surgery in right-sided colon cancer: (A) intra-abdominal abscess, (B) anastomotic bleeding, and (C) anastomotic leak. NOSE: Natural orifice specimen extraction.

In the pooled analysis of patients who underwent laparoscopic surgery for right-sided colon cancer, the NOSE group showed a significantly lower rate of SSI compared to the CVT group (OR 0.23; 95% CI: 0.08-0.73; p = 0.012; I² = 0%; Figure 2A), with minimal heterogeneity [3,8-13]. Similarly, the NOSE group demonstrated a significant reduction in postoperative pain scores on the third day (VAS score) (MD -1.1; 95% CI -1.7 to -0.5; p < 0.01; I² = 85.3%; Figure 2B) [8-11,13]. However, no significant difference was observed on the first postoperative day (MD -0.8; 95% CI -2.3 to 0.6; p = 0.26; I² = 95.9%; Figure 2C) [8-13]. High heterogeneity was noted in both VAS score outcomes. No statistically significant differences were found for other complications, including major complications graded as Clavien-Dindo ≥ III (OR 0.63; 95% CI 0.16-2.43; p = 0.503; I² = 0%; Figure 2D) [9,10,12], ileus (OR 0.51; 95% CI 0.19-1.37; p = 0.180; I² = 0%; Figure 3A) [3,8-10,12,13], urinary retention (OR 1.00; 95% CI 0.17-5.89; p = 1.000; I² = 0%; Figure 3B) [10-12], UTI (OR 1.15; 95% CI 0.21-6.26; p = 0.870; I² = 0%; Figure 3C) [8,9,13], intra-abdominal abscess (OR 0.89; 95% CI 0.22-3.61; p = 0.872; I² = 0%; Figure 4A) [3,8-10,12,13], anastomotic bleeding (OR 1.30; 95% CI 0.39-4.32; p = 0.671; I² = 0%; Figure 4B) [8-11,13], and anastomotic leak (OR 0.37; 95% CI 0.09-1.58; p = 0.181; I² = 0%; Figure 4C) [3,8-13]. All these outcomes demonstrated low heterogeneity.

Operative, Postoperative Outcomes, and Recurrence

**Figure 5:**
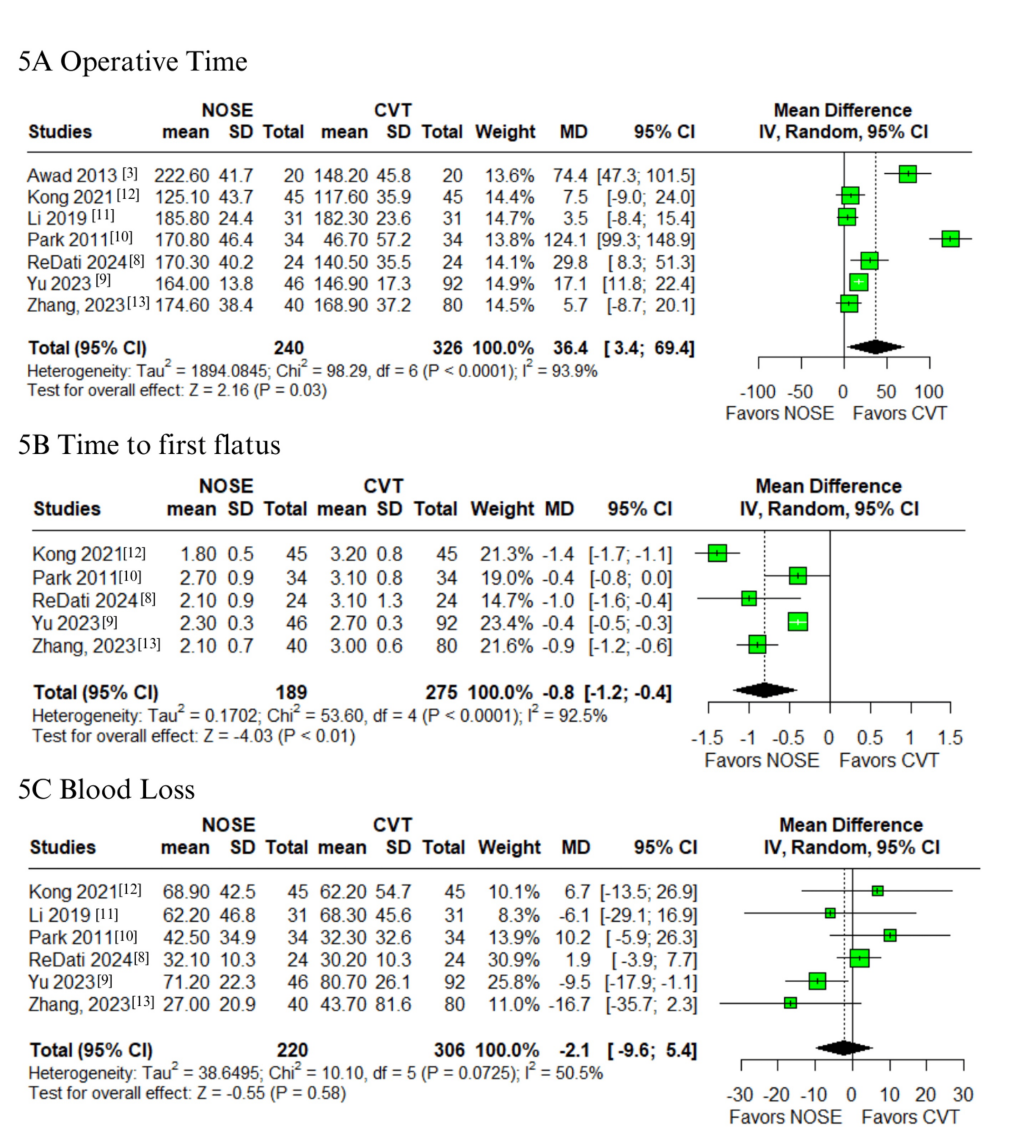
Forest plots comparing NOSE versus conventional laparoscopic surgery in right-sided colon cancer: (A) operative time, (B) time to first flatus, and (C) blood loss. NOSE: Natural orifice specimen extraction.

**Figure 6:**
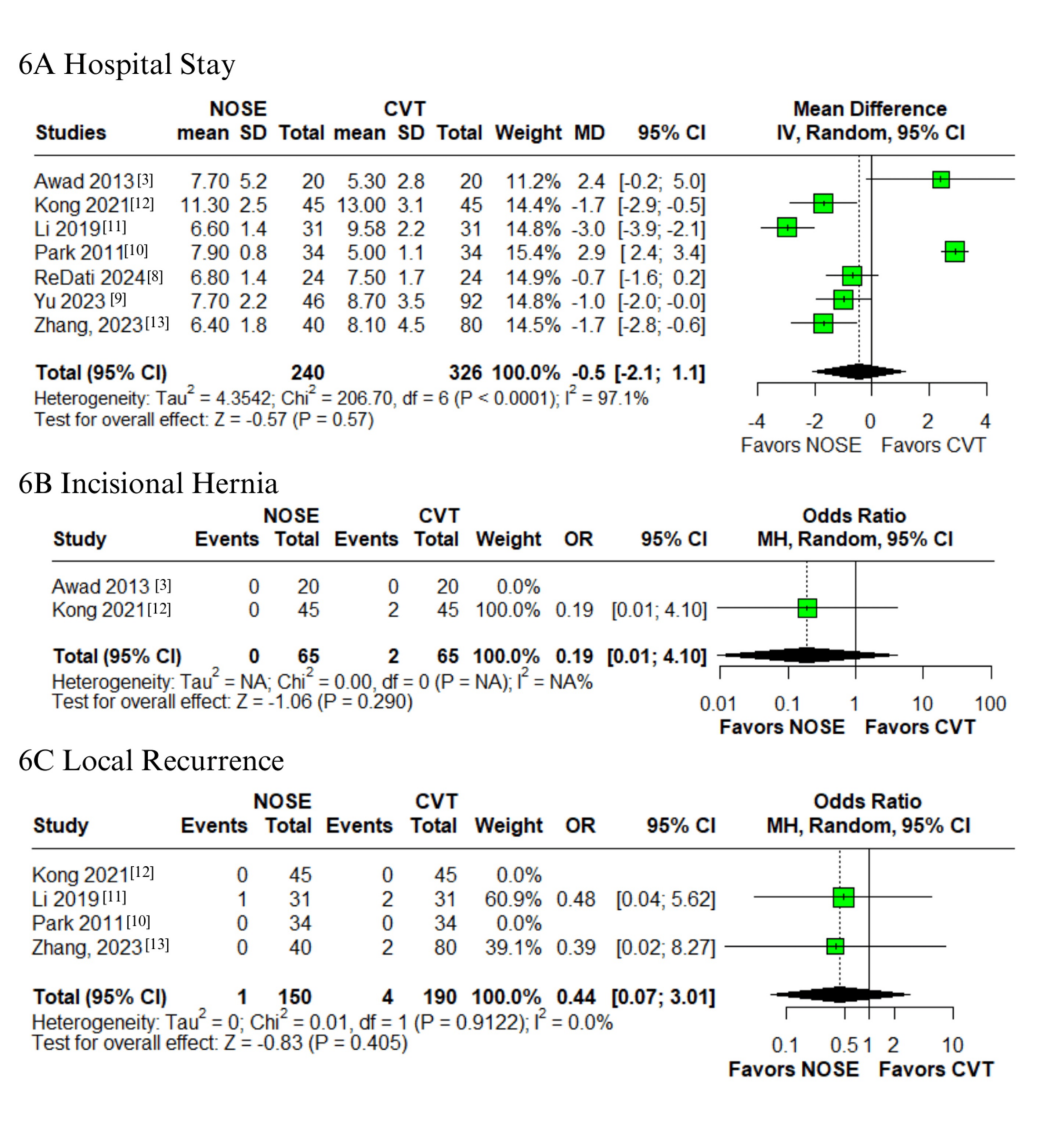
Forest plots comparing NOSE versus conventional laparoscopic surgery in right-sided colon cancer: (A) hospital stay, (B) incisional hernia, and (C) local recurrence. NOSE: Natural orifice specimen extraction.

Regarding operative and postoperative outcomes, the NOSE group was associated with a significantly longer operative time (MD 36.4 minutes; 95% CI 3.4-69.4; p = 0.03; I² = 93.9%; Figure 5A) [3,8-13]. However, time to first flatus was shorter in the NOSE group (MD -0.8; 95% CI -1.2 to -0.4; p < 0.01; I² = 92.5%; Figure 5B) [8-10,12,13]. No significant differences were found in intraoperative blood loss (MD -2.1; 95% CI -9.6 to 5.4; p = 0.58; I² = 50.5%; Figure 5C) or hospital stay (MD -0.5 days; 95% CI -2.1 to 1.1; p = 0.57; I² = 97.1%; Figure 6A) [3,8-13]. These outcomes exhibited high heterogeneity.

For long-term outcomes, the rates of incisional hernia (OR 0.19; 95% CI 0.01-4.10; p = 0.290; I² = NA; Figure 6B) and local recurrence (OR 0.44; 95% CI 0.07-3.01; p = 0.405; I² = 0%; Figure 6C) did not differ significantly between groups [3,10-13], and both outcomes demonstrated low or no heterogeneity.

Quality assessment

**Figure 7:**
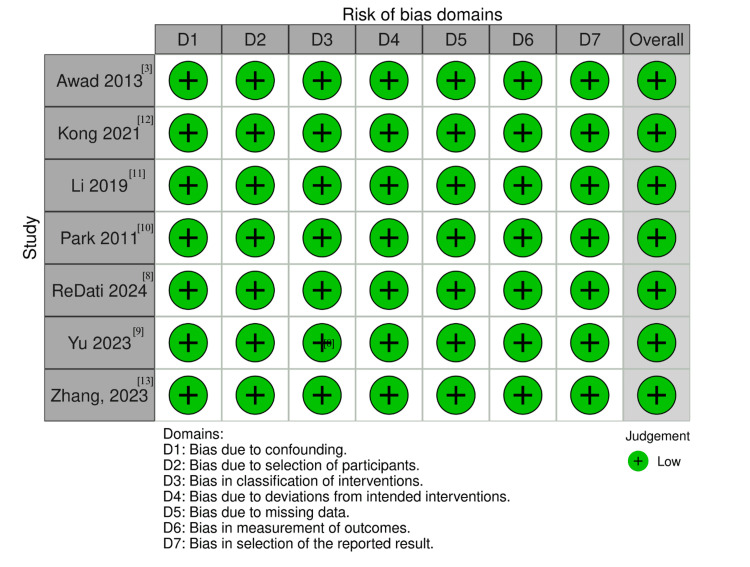
Critical appraisal of studies according to the Cochrane Collaboration’s tool for assessing risk of bias (ROBINS-I). ROBINS-I: Risk Of Bias In Non-randomized Studies of Interventions.

The individual risk of bias assessments for all included studies is presented in Figure 7. All observational studies were adjusted for confounding factors using PSM, which specifically addresses bias in Domain 1 of the ROBINS-I tool. A comprehensive evaluation across all seven ROBINS-I domains was conducted, and all studies were ultimately rated as having a low risk of bias.

Sensitivity analyses

**Figure 8:**
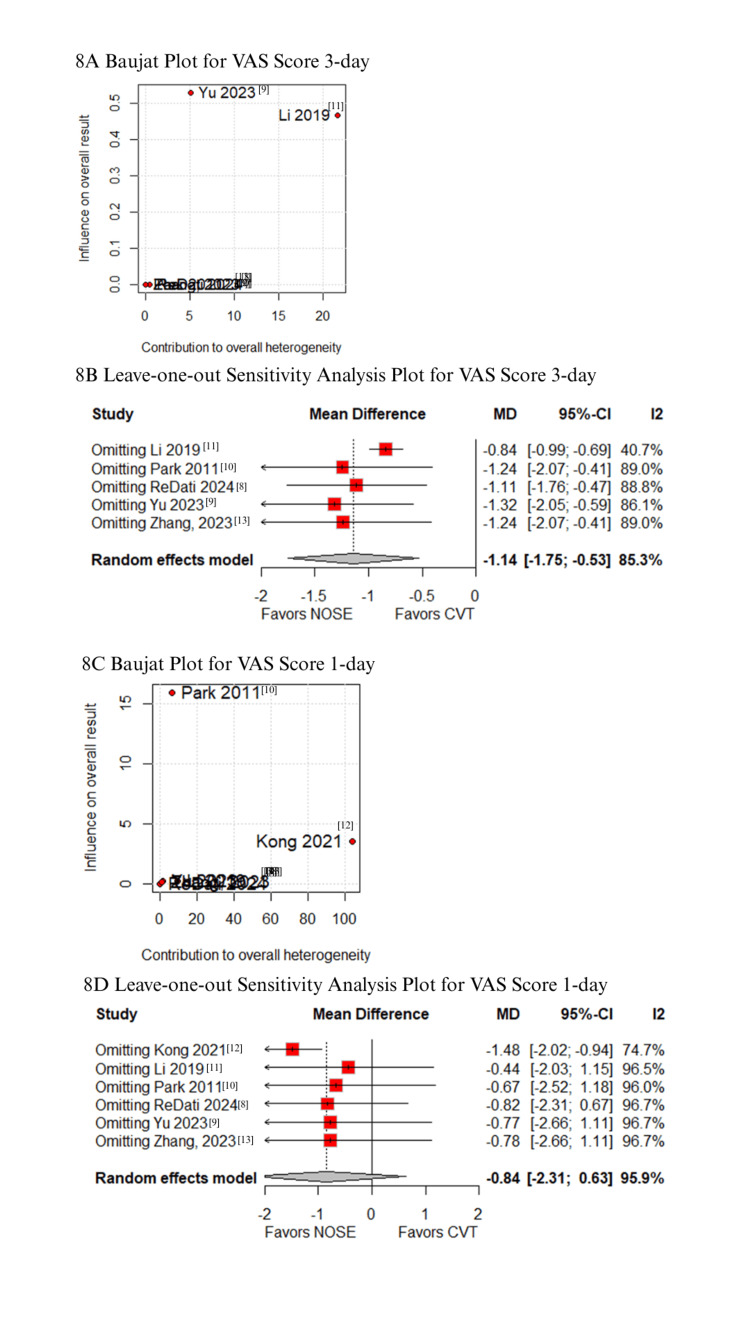
Sensitivity analyses: (A) Baujat plot for VAS score on postoperative day 3, (B) Leave-one-out plot for VAS score on postoperative day 3, (C) Baujat plot for VAS score on postoperative day 1, and (D) Leave-one-out plot for VAS score on postoperative day 1. VAS: Visual analog scale.

**Figure 9:**
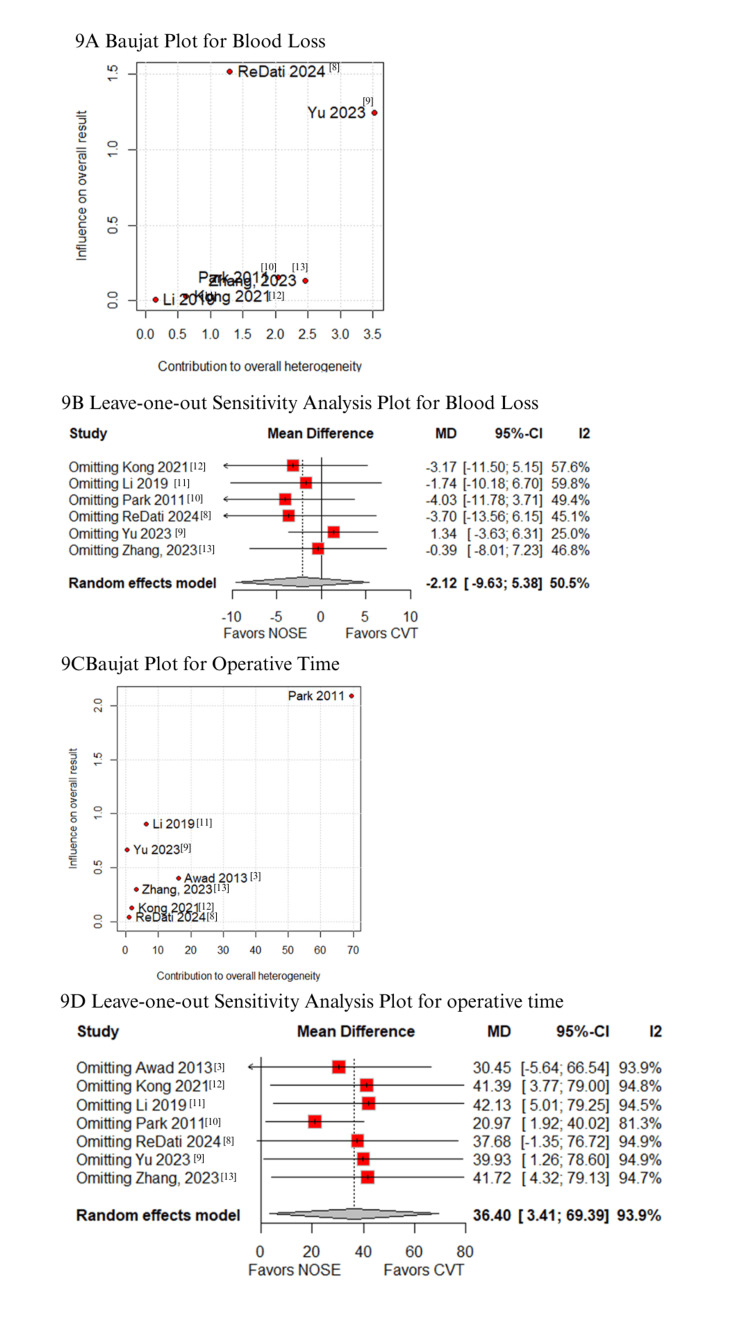
Sensitivity analyses: (A) Baujat plot for blood loss, (B) leave-one-out plot for blood loss, (C) Baujat plot for operative time, and (D) leave-one-out plot for operative time.

**Figure 10:**
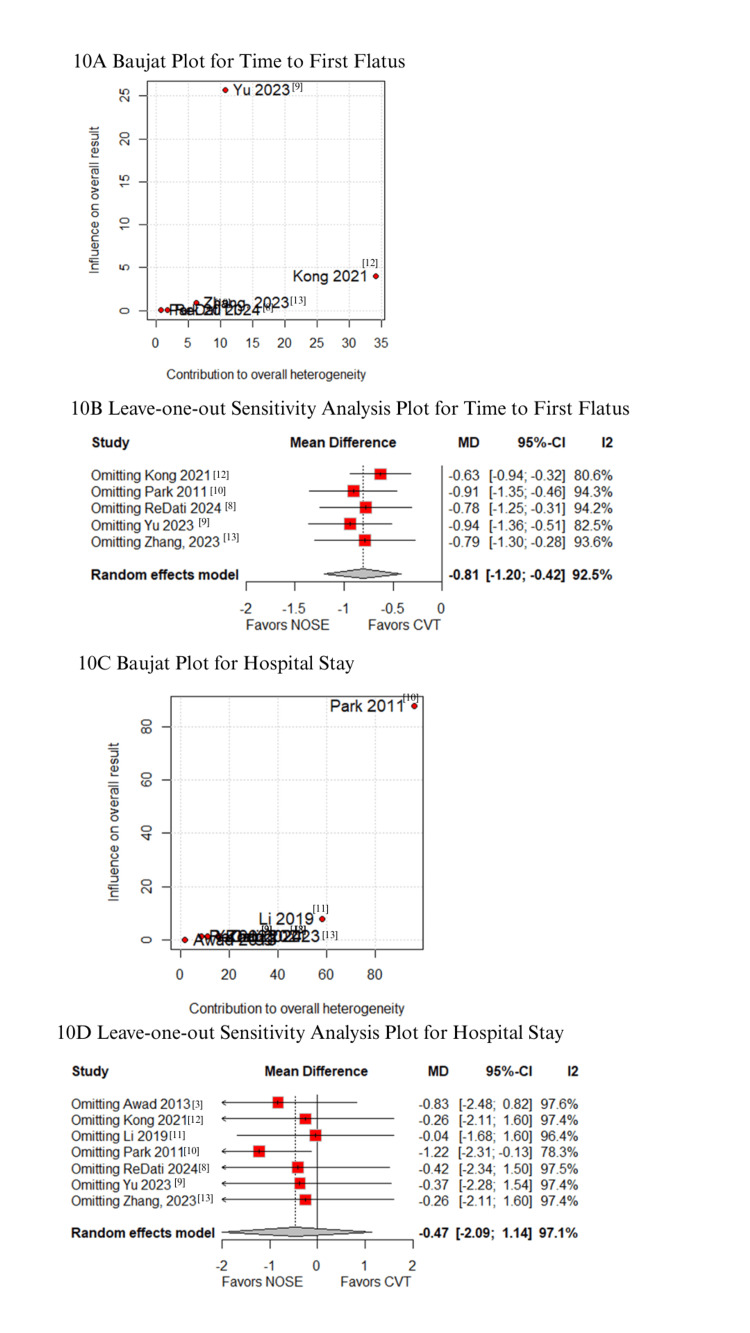
Sensitivity analyses: (A) Baujat plot for time to first flatus, (B) leave-one-out plot for time to first flatus, (C) Baujat plot for hospital stay, and (D) leave-one-out plot for hospital stay.

In the Baujat plot analysis, studies contributing most to heterogeneity were identified. For postoperative pain (VAS score on day 3), Li XW et al. was the primary contributor; however, its exclusion in the leave-one-out analysis did not alter the results (Figure 8A-8B) [11]. For VAS score on day 1, Kong FB et al. (2021) emerged as the major source of heterogeneity. Upon excluding this study, the results favored the NOSE group (MD -1.48; 95% CI -2.02 to -0.94; Figures 8C-8D) [12]. Regarding blood loss, Yu H et al. (2023) contributed the most to heterogeneity (Figures 9A-9B) [9], but leave-one-out analysis confirmed consistent results. Similarly, Park JS et al. (2011) was identified as the primary contributor to heterogeneity in operative time (Figures 9C-9D) [10]; exclusion of this study did not change the overall findings. For time to first flatus, Kong FB et al. (2021) again contributed most to heterogeneity, but the exclusion did not affect result consistency (Figures 10A-10D) [12]. Lastly, Park JS et al. (2011) was the main contributor to heterogeneity in hospital stay. Excluding this study significantly altered the results, favoring the NOSE group (MD -1.22; 95% CI -2.31 to -0.13; Figures 10C-10D) [10].

Discussion

This systematic review and meta-analysis, encompassing seven observational studies using PSM and a total of 566 patients undergoing laparoscopic surgery for right-sided colon cancer, revealed that the NOSE procedure was associated with significantly lower third-day VAS scores, faster passage of flatus, and reduced rates of SSI compared to conventional specimen extraction methods. After sensitivity analysis, first-day VAS scores and hospital stay duration also showed significant reductions in favor of the NOSE group. However, the NOSE group experienced longer operative times. No statistically significant differences were observed between the groups regarding severe complications (Clavien-Dindo ≥ III), ileus, urinary retention, UTIs, intra-abdominal abscesses, anastomotic bleeding, anastomotic leakage, intraoperative blood loss, incisional hernias, or local recurrence rates [3,8-13].

The advantages of conventional laparoscopic surgery over open approaches, such as reduced physical trauma and faster recovery, are well-established in modern clinical practice [20-25]. However, advancements in minimally invasive techniques have introduced scarless procedures like NOSE, which aim to further minimize wound complications, reduce postoperative discomfort, and enhance recovery outcomes [4,5,15,24-26]. First introduced by Franklin in 1993, NOSE enables specimen extraction through natural orifices, such as the vaginal, anal, or oral routes, eliminating the need for additional abdominal incisions [4,15,26-30]. Among these, transvaginal extraction has been the most widely utilized in right colectomies and has proven to be a safe and feasible alternative [3,8-13]. For instance, Zhang M et al. reported favorable oncologic outcomes and improved short-term recovery, with minimal impact on sexual function [31]. Similarly, Awad ZT and Griffin R reported transient dyspareunia in 5% of cases, which resolved within six months, while Park JS et al. observed no adverse effects on sexual function in their cohort [3,10]. Nonetheless, certain anatomical factors, such as large tumor size or thickened mesentery, may limit the feasibility of vaginal extraction in selected cases [3,10,31].

Although laparoscopic techniques have transformed colorectal surgery, specimen extraction still typically requires an abdominal incision, which can contribute to postoperative pain and delayed gastrointestinal recovery [4,15,26-29]. A RCT by Wolthuis AM et al. found that NOSE significantly reduced pain scores and analgesic requirements in patients undergoing surgery for colorectal cancer and diverticulitis compared to conventional extraction techniques [20]. Similarly, Chang SC et al. reported lower morphine consumption (33.9 mg vs. 43.4 mg; p = 0.011) and reduced postoperative discomfort in patients undergoing reduced-port laparoscopic procedures incorporating NOSE [27]. Furthermore, Cheng CC et al. and Gundogan E et al. have investigated NOSE in the context of right-sided colectomies, highlighting its effectiveness in pain reduction and enhanced recovery [6,7].

Recovery of bowel function was another key advantage of NOSE procedures [6,7]. Studies by Cheng CC et al. noted faster passage of flatus and first stool in NOSE patients, with statistical significance observed for the latter [6]. Our analysis further confirmed that NOSE facilitates quicker bowel recovery, although no significant difference was found in postoperative ileus rates. These findings align with previous reports highlighting the enhanced gastrointestinal recovery associated with NOSE procedures [4,15,26-30].

In contrast, operative time was prolonged in NOSE procedures compared to conventional approaches [3,8-13]. This disparity may be attributable to the learning curve associated with the technique, as well as the additional procedural steps required for natural orifice extraction. Some studies, such as those conducted by Cheng CC et al. and Gundogan E et al., reported no significant difference in operative times, but these were excluded from our analysis due to potential confounding biases, as we focused exclusively on PSM studies [6,7]. Despite heterogeneity in operative duration, sensitivity analyses reinforced the robustness of our results, emphasizing the need for further RCTs to validate these findings.

Our meta-analysis also identified lower SSI rates in the NOSE group, an important factor for postoperative wound healing [3,8-13]. The elimination of a laparotomy incision in NOSE procedures significantly reduces the likelihood of wound-related complications [4,5]. Even in comparative studies evaluating laparoscopic versus open techniques, extraction site complications remain a concern, particularly among obese patients [32-34]. For instance, Gundogan E et al. reported a 2.8% incidence of incisional hernias in conventional right colectomy cases [7]. In populations with higher obesity prevalence, NOSE may serve as an effective strategy to mitigate these complications and reduce healthcare costs [32-34]. In addition, a recent study identified low postoperative levels of butyrylcholinesterase (BChE) as an independent predictor of SSI after colorectal surgery. Reduced BChE concentrations on the first and third postoperative days were associated with more than a twofold increase in SSI risk, reinforcing its potential role as an early biomarker for infectious complications [35]. Although not yet implemented in clinical practice, BChE may offer a cost-effective adjunct for early identification of high-risk patients.

Lastly, concerns regarding the potential dissemination of malignant cells during NOSE procedures were not supported by our analysis. Neither NOSE for left-sided nor right-sided colon cancer showed increased recurrence rates or compromised oncologic outcomes [7,15,26-30]. Gundogan E et al. reported similar long-term results for NOSE and conventional approaches, with a mean follow-up of 27.4 ± 20.5 months [7]. Our results reinforce these conclusions, demonstrating equivalent local recurrence rates and affirming the long-term safety and short-term advantages of NOSE [7,15,26-30].

This study has several limitations that must be considered when interpreting the results. First, the meta-analysis exclusively included observational studies with propensity score matching, which, while reducing confounding biases, does not eliminate the potential for residual confounding inherent to non-randomized designs. Second, the high heterogeneity observed in some outcomes, particularly operative time and VAS scores, highlights the variability in surgical expertise, procedural standardization, and patient selection across the included studies. Third, the sample size of specific subgroups, such as patients undergoing transvaginal or transrectal specimen extraction, was limited, potentially affecting the generalizability of the findings to broader populations. Fourth, the inclusion of studies from predominantly Eastern populations, where obesity rates are lower, limits the applicability of these results to Western populations, where higher BMI may influence surgical outcomes and complication rates. Lastly, long-term oncological outcomes, such as disease-free survival and overall survival, were not consistently reported across studies, precluding a comprehensive analysis of NOSE's oncological safety. Future trials with larger sample sizes, more diverse patient populations, and standardized reporting of both short- and long-term outcomes are needed to address these limitations and further validate the benefits and risks of NOSE compared to conventional laparoscopic techniques.

## Conclusions

In conclusion, this systematic review and meta-analysis, encompassing 566 patients undergoing laparoscopic surgery for right-sided colon cancer, demonstrated that the NOSE procedure is associated with significantly lower third-day VAS scores, faster recovery of bowel function, and reduced SSI rates. Despite these benefits, operative times were notably longer for the NOSE procedure. Importantly, no significant differences were observed between the two approaches in terms of severe complications (Clavien-Dindo ≥ III), ileus, urinary retention, UTI, intra-abdominal abscess, anastomotic bleeding, leakage, intraoperative blood loss, incisional hernias, or local recurrence rates.

## Appendices

Appendix 1

**Table 3:** PRISMA 2020 checklist. Source: Reference [17]. For more information, visit: http://www.prisma-statement.org/

Section and Topic	Item #	Checklist item	Location where item is reported
Title			
Title	1	Natural Orifice Specimen Extraction for Right-Sided Colon Cancer: A Systematic Review and Meta-Analysis of Propensity Score-Matched Studies.	Page 1
Abstract			
Abstract	2	See the PRISMA 2020 for Abstracts checklist.	Page 3
Introduction			
Rationale	3	Describe the rationale for the review in the context of existing knowledge.	Par. 1-2
Objectives	4	Provide an explicit statement of the objective(s) or question(s) the review addresses.	Par. 3
Methods			
Eligibility criteria	5	Specify the inclusion and exclusion criteria for the review and how studies were grouped for the syntheses.	Pg. 5 Par. 3
Information sources	6	Specify all databases, registers, websites, organizations, reference lists, and other sources searched or consulted to identify studies. Specify the date when each source was last searched or consulted.	Pg. 6 Par. 1
Search strategy	7	Present the full search strategies for all databases, registers, and websites, including any filters and limits used.	Pg. 5 Par. 2
Selection process	8	Specify the methods used to decide whether a study met the inclusion criteria of the review, including how many reviewers screened each record and each report retrieved, whether they worked independently, and, if applicable, details of automation tools used in the process.	Pg. 6 Par. 1
Data collection process	9	Specify the methods used to collect data from reports, including how many reviewers collected data from each report, whether they worked independently, any processes for obtaining or confirming data from study investigators, and, if applicable, details of automation tools used in the process.	Pg. 6 Par. 1
Data items	10a	List and define all outcomes for which data were sought. Specify whether all results that were compatible with each outcome domain in each study were sought (e.g., for all measures, time points, analyses), and, if not, the methods used to decide which results to collect.	Pg. 6 Par. 1
Data items	10b	List and define all other variables for which data were sought (e.g., participant and intervention characteristics, funding sources). Describe any assumptions made about any missing or unclear information.	Pg. 6 Par. 1
Study risk of bias assessment	11	Specify the methods used to assess the risk of bias in the included studies, including details of the tool(s) used, how many reviewers assessed each study and whether they worked independently, and, if applicable, details of automation tools used in the process.	Pg. 6 Par. 2
Effect measures	12	Specify the effect measure(s) (e.g., risk ratio and mean difference) used in the synthesis or presentation of results for each outcome.	Pg. 6 Par. 3
Synthesis methods	13a	Describe the processes used to decide which studies were eligible for each synthesis (e.g., tabulating the study intervention characteristics and comparing against the planned groups for each synthesis (item #5)).	Pg. 6 Par. 3
Synthesis methods	13b	Describe any methods required to prepare the data for presentation or synthesis, such as handling of missing summary statistics or data conversions.	Pg. 6 Par. 3
Synthesis methods	13c	Describe any methods used to tabulate or visually display the results of individual studies and syntheses.	Pg. 6 Par. 3
Synthesis methods	13d	Describe any methods used to synthesize results and provide a rationale for the choice(s). If meta-analysis was performed, describe the model(s), method(s) to identify the presence and extent of statistical heterogeneity, and software package(s) used.	Pg. 6 Par. 3
Synthesis methods	13e	Describe any methods used to explore possible causes of heterogeneity among study results (e.g., subgroup analysis, meta-regression).	Pg. 6 Par. 3
Synthesis methods	13f	Describe any sensitivity analyses conducted to assess the robustness of the synthesized results.	Pg. 6 Par. 3
Reporting bias assessment	14	Describe any methods used to assess the risk of bias due to missing results in a synthesis (arising from reporting biases).	Pg. 6 Par. 2
Certainty assessment	15	Describe any methods used to assess certainty (or confidence) in the body of evidence for an outcome.	Pg. 6 Par. 2
Results			
Study selection	16a	Describe the results of the search and selection process, from the number of records identified in the search to the number of studies included in the review, ideally using a flow diagram.	Figure 1
Study selection	16b	Cite studies that might appear to meet the inclusion criteria, but which were excluded, and explain why they were excluded.	Figure 1
Study characteristics	17	Cite each included study and present its characteristics.	Table 1 / 2
Risk of bias in studies	18	Present assessments of risk of bias for each included study.	Figure 16
Results of individual studies	19	For all outcomes, present, for each study: (a) summary statistics for each group (where appropriate) and (b) an effect estimate and its precision (e.g., confidence/credible interval), ideally using structured tables or plots.	Figures 2-5
Results of syntheses	20a	For each synthesis, briefly summarise the characteristics and risk of bias among contributing studies.	Figure 6
Results of syntheses	20b	Present results of all statistical syntheses conducted. If meta-analysis was done, present for each the summary estimate and its precision (e.g., confidence/credible interval) and measures of statistical heterogeneity. If comparing groups, describe the direction of the effect.	Pg. 7-8
Results of syntheses	20c	Present results of all investigations of possible causes of heterogeneity among study results.	Pg. 12 Par. 1
Results of syntheses	20d	Present results of all sensitivity analyses conducted to assess the robustness of the synthesized results.	Pg. 12 Par. 1
Reporting biases	21	Present assessments of risk of bias due to missing results (arising from reporting biases) for each synthesis assessed.	Pg. 12 Par. 2
Certainty of evidence	22	Present assessments of certainty (or confidence) in the body of evidence for each outcome assessed.	Pg. 12 Par. 1
Discussion			
Discussion	23a	Provide a general interpretation of the results in the context of other evidence.	Pg. 12 Par. 1
Discussion	23b	Discuss any limitations of the evidence included in the review.	Pg. 14 Par. 1
Discussion	23c	Discuss any limitations of the review processes used.	Pg. 14 Par. 1
Discussion	23d	Discuss implications of the results for practice, policy, and future research.	Pg. 14 Par. 1
Other Information			
Registration and protocol	24a	Provide registration information for the review, including register name and registration number, or state that the review was not registered.	Pg. 5 Par. 1
Registration and protocol	24b	Indicate where the review protocol can be accessed, or state that a protocol was not prepared.	Pg. 5 Par. 1
Registration and protocol	24c	Describe and explain any amendments to information provided at registration or in the protocol.	Pg. 5 Par. 1
Support	25	Describe sources of financial or non-financial support for the review, and the role of the funders or sponsors in the review.	Pg. 2
Competing interests	26	Declare any competing interests of review authors.	Pg. 2
Availability of data, code, and other materials	27	Report which of the following are publicly available and where they can be found: template data collection forms; data extracted from included studies; data used for all analyses; analytic code; any other materials used in the review.	Supplementary
